# Helping to Feel Better or Do Better? Implications of Interpersonal Emotion Regulation for Emotional Well-Being and Successful Goal Pursuit

**DOI:** 10.3390/bs16071071

**Published:** 2026-06-30

**Authors:** Lisanne S. Pauw, Laura Kirin, Francesca Righetti

**Affiliations:** 1 Department of Social, Health and Organisational Psychology, Utrecht University, 3584 CS Utrecht, The Netherlands; 2 Department of Experimental and Applied Psychology, VU University, 1081 HV Amsterdam, The Netherlands; laura.kirin@gmail.com (L.K.); f.righetti@vu.nl (F.R.)

**Keywords:** interpersonal emotion regulation, regulatory flexibility, personal growth, goal pursuit, well-being

## Abstract

Personal growth often requires people to cope with setbacks and the negative emotions they evoke. Romantic partners can serve as important sources of support in these situations. In this research, we examined which interpersonal emotion regulation (IER) strategies are most effective for supporting two common goals in such contexts: improving emotional well-being and facilitating goal pursuit. Across three studies, we investigated the effectiveness of seven IER strategies (i.e., interpersonal acceptance, reappraisal, problem-solving, co-rumination, distraction, suppression and ignoring) for helping one’s partner feel better (hedonic goal) vs. achieve their goal (instrumental goal). Study 1 assessed whether the perceived effectiveness of these IER strategies was dependent on people’s hedonic vs. instrumental regulatory goal. Study 2 examined naturally occurring IER in romantic relationships, testing which IER strategies were related to perceived efficacy of the partner in fostering hedonic and instrumental goal achievement. Study 3 used a one-month longitudinal design to examine changes in negative affect and goal progress over time. Results showed that while people perceive IER strategies as differentially effective for hedonic versus instrumental goals, strategies that address obstacles (i.e., problem-solving) may be especially effective in helping romantic partners support both emotional well-being and goal pursuit, two processes central to personal growth.

## 1. Introduction

A key feature of adaptive functioning is the capacity to actively shape one’s own development over time (Brunstein et al., 1999; Kashdan & Rottenberg, 2010). Personal growth captures this capacity by reflecting people’s efforts to pursue goals, take actions, and regulate their emotions adaptively in response to situational demands (Bobinet et al., 2025; Brandstätter & Bernecker, 2022). As such, personal growth has been argued to constitute a central component of psychological well-being (Ryff, 2014). Although personal growth can follow major life events (Bauer & McAdams, 2004; Mangelsdorf & Eid, 2015), it frequently unfolds through everyday efforts to pursue meaningful goals and cope with setbacks and its associated emotional challenges (Bobinet et al., 2025; Ganti et al., 2025). Because these efforts rarely occur in isolation, close relationships—and romantic partners in particular—may play a central role in fostering personal growth by helping one other regulate emotions and pursue important goals through interpersonal emotion regulation.

Interpersonal emotion regulation (IER) includes attempts by one person to influence the emotions of another (Niven, 2017; Zaki & Williams, 2013), and it has been recognized as a core relational process that impacts emotional and relational well-being of both partners (Arican-Dinc & Gable, 2025; Bodenmann, 2005; Butler & Randall, 2013). Recent studies distinguish among the strategies people use to regulate others’ emotions (e.g., Kneeland et al., 2024; MacCann et al., 2025; Swerdlow & Johnson, 2022). Strategies such as interpersonal reappraisal, acceptance, and collaborative problem-solving are generally associated with more adaptive outcomes, whereas strategies including interpersonal suppression, ignoring, or co-rumination tend to predict poorer emotional and relational outcomes (Jurkiewicz et al., 2023; MacCann et al., 2025; Pauw et al., 2024; Rose et al., 2014; Sahi et al., 2025). However, this work has largely treated the effectiveness of these strategies as relatively stable across contexts, thereby giving limited attention to the contextual conditions under which their consequences may vary.

Importantly, recent theoretical accounts emphasize that while some emotion regulation strategies may be overall associated with more adaptive outcomes than others, their effectiveness is fundamentally context-dependent: whether a strategy is beneficial depends on the goal it is intended to serve and the situational demands at hand (Aldao et al., 2015; Bonanno & Burton, 2013; Kashdan & Rottenberg, 2010). In this regard, a central distinction concerns regulating emotions for hedonic reasons (i.e., to feel better) or for instrumental reasons (i.e., to make progress towards a certain goal; Tamir, 2009; Tamir & Millgram, 2017). While a growing body of evidence suggests that the effectiveness of emotion regulation strategies is goal-dependent in the intrapersonal domain (i.e., when people regulate their own emotions; Kobylińska & Kusev, 2019), these theoretical assumptions remain untested in the interpersonal domain. That is, it remains unclear whether the IER strategies enacted by a partner differ in their effectiveness as a function of the target’s underlying goal. The present research addresses this gap by examining the goal-dependent effectiveness of IER strategies. Specifically, we aim to (a) identify which strategies are perceived as effective within hedonic and instrumental contexts and (b) test whether their perceived effectiveness differs across these types of goals. As such, we aim to contribute to a clearer understanding of which IER strategies are perceived as helpful for different goals, providing insight into how close relationships may support emotional well-being and personal growth over time.

### 1.1. The Role of Emotion Regulation in Personal Growth

Although definitions of personal growth vary across disciplines (Ganti et al., 2025), many agree on the importance of striving to improve oneself through successfully overcoming challenges (Bobinet et al., 2025). Rather than being a fixed end state, personal growth is increasingly conceptualized as an intentional, ongoing process of development that involves adapting to setbacks and continuously changing circumstances (Bobinet et al., 2025). Because growth-relevant goals are often difficult and prolonged, this process is inherently demanding and often elicits negative emotions such as sadness, anxiety or frustration, particularly when obstacles arise or progress is slower than anticipated (Carver & Scheier, 1998, 2011; Szumowska et al., 2025). When these emotions become too intense, they can be experienced as discouraging and undermine goal pursuit (Thompson, 2019). Effective emotion regulation should therefore play a central role in fostering personal growth. By managing emotional responses to setbacks, people can stay motivated and committed to their goal, and remain open to revising strategies when needed (Benita et al., 2024; Carver & Scheier, 2011). Successfully coping with these emotional experiences is thus not only critical for maintaining well-being in the moment, but also for supporting the adaptive self-regulation processes that enable individuals to develop personally over time (Bobinet et al., 2025).

Close others can play a central role in these processes, as emotion regulation is effortful and often requires social support (Levy-Gigi & Shamay-Tsoory, 2017; Reeck et al., 2016; Williams et al., 2018). Romantic partners can be an essential source of support: By helping the other accomplish meaningful goals and improve their emotional well-being, partners can help each other grow, which in turn promotes flourishing at the individual and relational level (Feeney & Collins, 2015; Fitzsimons et al., 2015; Rusbult et al., 2009). Such support can take the form of interpersonal emotion regulation (IER; Niven, 2017; Zaki & Williams, 2013). Importantly, people may try to regulate others’ emotions through a variety of different strategies, which may be associated with differential outcomes. A key question, therefore, is which IER strategies are most effective in fostering goal progress and emotional well-being.

*Interpersonal acceptance* involves validating the other’s emotions without judging them or trying to change them (Hayes, 2004; Jurkiewicz et al., 2023). It is typically perceived as comforting, and has positive relational outcomes (MacCann et al., 2025; Pauw et al., 2018; Sahi et al., 2023), though support for its effectiveness in downregulating negative affect is more mixed (Batenburg & Das, 2014; Jurkiewicz et al., 2023; Pauw et al., 2024). *Interpersonal (or social) reappraisal* involves helping the other reframe the situation to change its emotional impact, and has generally been associated with improved emotional and relational outcomes (Jurkiewicz et al., 2023; MacCann et al., 2025; Sahi et al., 2023, 2025, though see Pauw et al., 2024 for null findings). *Co-rumination* entails excessively talking about problems and dwelling on negative feelings, which has been associated with worsened emotional outcomes, though positive relational consequences (Rose, 2021). *Problem-solving* constitutes identifying solutions to help the other change or control the situation (MacGeorge et al., 2004). When effective in resolving the stressor, it can reduce the associated distress and improve relational well-being by signaling emotional involvement (Aldao et al., 2010; Morelli et al., 2015; Semmer et al., 2008). On the other hand, it may also cause reactance and impede well-being when it takes the form of unsolicited advice (Pauw et al., 2024; Riccioni et al., 2014).

In contrast to the strategies described above, *interpersonal distraction* aims to take the attention away from the emotional event, which has been associated with enhanced emotional and relational outcomes—though these effects are typically short-lived, as distraction does not allow processing of the emotional event (MacCann et al., 2025; Pauw et al., 2024; Ruan et al., 2024). *Interpersonal suppression* involves encouraging the other not to feel, express or think about their emotional experience, and has been associated with impaired emotional and relational outcomes (Little et al., 2013; Pauw et al., 2024, though see Ruan et al., 2024, for opposite findings). Finally, although not classically studied as an IER strategy, *ignoring* the other’s emotions (i.e., the absence of regulation) mirrors experiential avoidance of one’s own emotions, and is associated with worse emotional and relational outcomes (Aldao et al., 2010; Pauw et al., 2024).

### 1.2. Goal-Dependent Interpersonal Emotion Regulation

While much of the existing IER literature implicitly treats these strategies as inherently adaptive or maladaptive, recent theoretical accounts have called for a more context-sensitive approach, arguing that IER effectiveness should be assessed in relation to the regulatory goals that it is meant to serve (Dixon-Gordon et al., 2015). In fact, growing evidence from research on intrapersonal emotion regulation shows that people regulate emotions not only to maximize pleasure and minimize distress, but also to support goal achievement (Tamir, 2009; Tamir & Millgram, 2017). Therefore, a key distinction can be made between regulating emotions for *hedonic* motives, which refer to regulating emotions to improve subjective feeling states, such as reducing sadness or anxiety, and *instrumental* motives, which refer to regulating emotions as a means to an end, such as to facilitate performance, navigate social interactions or foster personal growth (Niven, 2016; Tamir, 2016). Importantly, even though previous research has mostly focused on hedonic motives, experience-sampling research suggests that instrumental motives are also frequently endorsed in daily life, with performance-related motives being most prevalent (Kalokerinos et al., 2017).

The distinction between hedonic and instrumental motives is particularly relevant in the context of everyday goal pursuit, given that what counts as “effective” emotion regulation may depend on whether the immediate aim is emotional relief or goal achievement. Research on intrapersonal emotion regulation shows that people are sometimes willing to experience negative emotions when they believe these emotions may facilitate goal attainment (Tamir, 2009; Tamir & Millgram, 2017). Accordingly, strategies that are often considered maladaptive may serve useful functions in certain contexts (Aldao, 2013; Sheppes, 2020). For example, expressive suppression can be adaptive when hiding one’s emotional reaction facilitates the goal of maintaining social harmony or avoiding conflict (Bonanno et al., 2004). Similarly, while distraction (unlike reappraisal) may not serve long-term emotional recovery, it might be more effective in situations that call for immediate relief, such as when task engagement is required (cf. Sheppes et al., 2014; Sheppes & Meiran, 2008). These findings highlight that the effectiveness of emotion regulation strategies depends not only on the strategy itself but also on the goals and situational demands they are intended to serve.

Consistent with this idea, research on intrapersonal emotion regulation shows that hedonic and instrumental motives shape the strategies that people select to regulate their own emotions. Work by English and colleagues has shown that people flexibly use different regulatory strategies depending on their goals. For example, people use more suppression—a strategy typically viewed as maladaptive—when regulating their emotions for instrumental (rather hedonic) reasons, such as for impression management or avoiding conflict (Eldesouky & English, 2019; English et al., 2017). 

Likewise, recent work has shown that people are also context-sensitive in selecting different regulatory strategies when trying to regulate *others*’ emotions. Stronger hedonic motives (i.e., trying to make the other feel better) have been associated with a greater use of interpersonal acceptance and reappraisal, and a decreased use of interpersonal suppression (Jurkiewicz et al., 2023). Moreover, people seem to provide more emotional support when a target appears to seek emotional relief (i.e., is in a situation implying a hedonic goal), whereas they engage in more interpersonal suppression and distraction when the target needs to perform well (i.e., is in a situation implying an instrumental goal; Pauw et al., 2019). These findings suggest that people choose different strategies to regulate others’ emotions depending on whether hedonic or instrumental goals are salient. Importantly, however, it remains unclear whether these strategies differ in their effectiveness as a function of the target’s regulatory goal. That is, we do not yet know whether certain IER strategies are more or less beneficial depending on whether the target seeks immediate emotional relief or progress towards an instrumental goal.

### 1.3. The Present Research

The present research aims to advance understanding of how close relationships may facilitate adaptive functioning and personal growth by examining which IER strategies are effective for hedonic and instrumental goals and whether their effectiveness depends on the type of goal. In doing so, we respond to calls for a more context-sensitive approach to IER, which emphasize that the effectiveness of regulatory efforts should not only be evaluated in terms of affective improvement, but also by whether they support the goals people are pursuing (Aldao & Tull, 2015; Dixon-Gordon et al., 2015). Although prior research has often characterized IER strategies as broadly adaptive or maladaptive based on their hedonic outcomes (Dixon-Gordon et al., 2015), everyday regulation frequently serves instrumental purposes as well (Kalokerinos et al., 2017; Tamir, 2016). Importantly, we are unaware of empirical studies testing the effectiveness of common IER strategies in promoting goal progress. This may be particularly relevant in the context of personal growth, which involves sustained goal pursuit when confronted with setbacks and emotional challenges (Bobinet et al., 2025).

Accordingly, we distinguish between hedonic and instrumental regulatory goals and test whether IER strategies differ in their effectiveness across these contexts. First, we sought to identify which IER strategies (interpersonal acceptance, reappraisal, distraction, suppression, problem-solving, co-rumination, and ignoring) are effective when targets pursue hedonic versus instrumental goals. Second, we examined whether the effectiveness of these strategies varied as a function of the regulatory goal—that is, whether certain strategies are differentially effective depending on whether the target seeks emotional relief or goal-directed progress.

Across three studies, we used complementary methods to address these questions. Study 1 adopted an experimental design, examining whether targets perceive seven IER strategies as differentially effective depending on when they have a hedonic vs. instrumental goal. Study 2 involved a cross-sectional survey, testing associations between targets’ reports of their partner’s typical IER strategy use and efficacy in achieving both regulatory goals. Study 3 employed a longitudinal design to test to what extent IER strategy use by the partner predicted actual progress in both regulatory goals over time (i.e., reduced negative affect and enhanced goal progress). By investigating both the absolute effectiveness of strategies within each goal context and their relative effectiveness across goals, the present set of studies aimed to contribute to a more context-sensitive understanding of IER, as well as provide insight into how close relationships can support adaptive goal pursuit and personal growth over time.

## 2. Study 1

In Study 1, we examined whether targets perceive IER strategies as differentially effective depending on their regulatory goal. Specifically, we differentiated between hedonic goals (i.e., wanting to feel better) vs. performance-oriented instrumental goals (e.g., wanting to get work done). Participants were randomly assigned to one of the two regulatory goal conditions and then rated the effectiveness of seven IER strategies (interpersonal acceptance, reappraisal, distraction, suppression, problem-solving, co-rumination, and ignoring) in relation to this specific goal. Overall, we hypothesized that these IER strategies would be perceived as differentially effective depending on targets’ regulatory goal. More specifically, given that interpersonal reappraisal and acceptance are typically effective in down-regulating negative affect (e.g., Jurkiewicz et al., 2023), though do require people to emotionally and cognitively engage with the emotional experience (MacCann et al., 2025), we hypothesized that these strategies would be perceived as more effective by targets who have a hedonic goal compared to targets who have an instrumental goal (cf. Pauw et al., 2019). In contrast, we hypothesized that strategies focused on disengaging from the emotional experience (i.e., interpersonal suppression, distraction and ignoring) or on removing obstacles to goal pursuit (i.e., problem-solving) would be perceived as more effective by targets who have an instrumental goal compared to targets who have a hedonic goal (cf. Pauw et al., 2019). Finally, given the mixed outcomes typically associated with co-rumination (Rose, 2021), we made no predictions regarding the effect of targets’ regulatory goal on the perceived effectiveness of co-rumination. We preregistered our hypotheses on OSF (see https://osf.io/bw92c/overview?view_only=dbc731e48efd42498101dfc6b32a8fc3, accessed on 18 April 2024).

### 2.1. Materials and Methods

#### 2.1.1. Participants

Participants were recruited using Prolific. Eligibility criteria included being 18 years or older, being fluent in English, and being in a romantic relationship for at least six months. An a priori power analysis was conducted using G*Power version 3.1.9.6 (Faul et al., 2007) to determine the necessary sample size to detect a within-between subjects interaction effect in a mixed ANOVA (2 groups × 7 measurements). Assuming a small-to-medium effect size (Cohen’s *f* = 0.15), desired power of 0.95, and an alpha rate of .05, 930 participants were required. To allow for potential dropout, we recruited a total of 1017 participants. Participants who failed two or more (out of four) attention checks or indicated at the end of the study that their responses should be discarded because they had responded randomly or dishonestly were excluded from the analyses (*N* = 14). After filtering for eligibility and attention checks, 993 participants were included in the analyses, aged between 20 and 78 (*M* = 44.1, *SD* = 13.1), with 62.6% identifying as female and 36.5% as male. The majority of the participants identified as White (90.0%), with the remainder of the sample identifying as Asian/Asian British/Asian Welsh (4.8%), Black/Black British/Black Welsh/Caribbean or African (2.2%), Mixed/Multiple ethnic groups (2.1%) or Other ethnic group (0.5%). Average relationship duration varied between 8 months and 61 years (*M* = 17.1 years, *SD* = 12.5 years). The majority (88.8%) lived with their partner, 67.8% were married or engaged, and 60.7% had children.

#### 2.1.2. Procedure

The study was approved by the Ethics Review Board of the Faculty of Social and Behavioral Sciences of Utrecht University, the Netherlands. After giving informed consent, participants were asked about their relationship status and duration, which determined their eligibility. If eligible, participants were randomly assigned to the instrumental goal vs. hedonic goal condition, which was manipulated using a prompt. Then, they rated how helpful they would find seven IER strategies when enacted by their partner, in relation to their assigned regulatory goal. Afterwards, we administered a manipulation check to ensure the manipulation was successful. Next, they answered several questionnaires unrelated to the present study purpose (i.e., relationship satisfaction, well-being, attachment and typical use of co-rumination). Lastly, they answered demographic questions and an honesty check. At the end of the study, participants were debriefed, and financially compensated with an hourly wage of £8 if they passed the required attention checks.

#### 2.1.3. Measures

**IER Goal Manipulation:** To manipulate the (hedonic vs. instrumental) regulatory goal, participants first received a brief explanation of interpersonal emotion regulation. They were then asked to think either about “moments when you’re upset and want to feel better” (hedonic goal) or “moments when you’re upset and want to get work done” (instrumental goal) in a between-subjects design. Next, participants rated the extent to which they found it helpful when their partner responded in various ways (tapping into seven different IER strategies). Specifically, they were asked: “When I want to feel better|When I want to get work done, I find it helpful when my partner”, followed by the strategies outlined below (for the full prompts, see Supplementary Section S1).

**Perceived Effectiveness of IER Strategies:** Seven IER strategies were assessed using three items per strategy, presented in random order. Items were taken and adapted from prior studies (Jurkiewicz et al., 2023; Pauw et al., 2024; Rose et al., 2014; Ruan et al., 2024; Swerdlow & Johnson, 2022). Item selection was guided by conceptual fit with each strategy and face validity. Because existing items were often developed for different contexts, wording was adapted to reflect interpersonal regulation within the goal-directed contexts used in the present study. When no suitable items were available, additional items were developed to ensure adequate coverage of each strategy and to maintain a parallel structure across the scale. Participants rated the perceived helpfulness of each strategy in relation to the regulatory goal to which they had been assigned on a 7-point Likert scale (1 = completely disagree, 7 = completely agree). The strategies included co-rumination (e.g., “repeatedly engages me in conversation about my negative emotions,” α = .64), interpersonal suppression (e.g., “encourages me to keep my emotions to myself,” α = .58), interpersonal distraction (e.g., “tries to direct my attention to something else,” α = .75), interpersonal reappraisal (e.g., “tries to make me think differently about the situation,” α = .80), interpersonal acceptance (e.g., “expresses that I should let myself feel the emotions and accept them,” α = .78), interpersonal problem-solving (e.g., “helps me to make a plan,” α = .82), and interpersonal ignoring (e.g., “does not get involved in the situation,” α = .78). A confirmatory factor analysis (CFA) was generally consistent with proposed seven-factor structure of IER strategies (see Supplementary Section S2 for more details). Accordingly, an average score was computed per strategy, with higher scores thus reflecting greater perceived effectiveness of the strategy.

**Manipulation Check:** To assess the effectiveness of the manipulation, participants indicated the extent to which they had thought about the intended type of situation (i.e., regulatory goal) while rating the effectiveness of the IER strategies. Specifically, they rated their agreement with the following statements: “When answering the questions on the previous page, I tried to think of situations in which I’m upset and want to feel better” and “When answering the questions on the previous page, I tried to think of situations in which I’m upset and want to get work done.” Responses were provided on a 7-point Likert scale (1 = completely disagree, 7 = completely agree).

### 2.2. Results

#### 2.2.1. Manipulation Check

To test whether the manipulation was successful, we ran a 2 (between-subjects: hedonic vs. instrumental goal condition) × 2 (within-subjects: self-reported hedonic vs. instrumental goal) mixed ANOVA. There was a significant interaction between Regulatory Goal Condition x Self-Reported Goal, *F*(1, 991) = 465.79, *p* < .001, η_p_^2^ = .32. As intended, those in the hedonic goal condition reported a stronger hedonic goal (*M* = 6.14, *SD* = 0.95) compared to those in the instrumental goal condition (*M* = 5.19, *SD* = 1.73; *B* = 0.96, *t*[1, 991] = 10.76, *p* < .001), whereas those in the instrumental goal condition reported a stronger instrumental goal (*M* = 5.97, *SD* = 1.09) compared to those in the hedonic goal condition (*M* = 4.19, *SD* = 1.79, *B* = −1.78, *t*[1, 991] = −18.94, *p* < .001). Moreover, within the hedonic goal condition, participants had a stronger hedonic rather than instrumental goal in mind (*p* < .001), whereas within the instrumental goal condition, participants had a stronger instrumental rather than hedonic goal in mind (*p* < .001). Together, these findings indicate that the manipulation of regulatory goal was successful.

#### 2.2.2. Goal-Dependent IER Strategy Effectiveness

To test whether IER strategies were perceived as differentially effective depending on the regulatory goal, we ran a 2 (between-subjects: Regulatory Goal Condition) × 7 (within-subjects: IER Strategy) Repeated Measures ANOVA. Mauchly’s test indicated that the assumption of sphericity was violated, χ^2^(20) = 1283.10, *p* < .001. Therefore, Greenhouse–Geisser corrections were applied. All means and standard deviations can be found in Table 1. Correlations among strategies can be found in Table S1 in the Supplementary Materials. There was a significant main effect of Regulatory Goal Condition (*F*[1, 991] = 5.21, *p* = .023, η_p_^2^ = .01), indicating that overall, IER strategies were perceived as slightly more effective for the hedonic goal (*M* = 4.26, *SD* = 0.55) than the instrumental goal (*M* = 4.18, *SD* = 0.61). Moreover, we found a significant main effect of IER Strategy (*F*[3.85, 3811.78] = 1283.55, *p* < .001, η_p_^2^ = .56), indicating that there was an overall difference in perceived effectiveness of the IER strategies, regardless of the goal (see Table 1 for pairwise comparisons among the total means). Interpersonal acceptance, reappraisal and problem-solving were perceived as the most helpful strategies across both regulatory goals. The perceived helpfulness of the other four strategies varied more across regulatory goals, with interpersonal distraction and co-rumination being rated as relatively helpful (above midpoint of the scale), and interpersonal suppression and ignoring being rated as relatively unhelpful (below midpoint of the scale).

Furthermore, and in line with our overarching hypothesis, there was a significant interaction between Regulatory Goal Condition and IER Strategies (*F*[3.85, 3811.78] = 36.15, *p* < .001, η_p_^2^ = .04), indicating the perceived effectiveness of the IER strategies was dependent on the regulatory goal. Follow-up Bonferroni-corrected pairwise comparisons (see Table 1) indicated that interpersonal acceptance, distraction and co-rumination were perceived as more effective for the hedonic goal than for the instrumental goal. In contrast, interpersonal suppression, and ignoring were perceived as more effective for the instrumental goal than the hedonic goal. Finally, reappraisal and problem-solving did not differ significantly in perceived effectiveness between the two regulatory goal conditions.

### 2.3. Discussion

In Study 1, we recruited participants who were in a relationship and asked them how helpful they would find it if their partner would use different IER strategies in the context of their randomly assigned regulatory goal. Overall, participants reported interpersonal reappraisal, acceptance and problem-solving as most helpful, and interpersonal suppression and ignoring as least helpful. Importantly, the perceived effectiveness of several strategies was goal dependent. Specifically, interpersonal acceptance, distraction and co-rumination were considered as relatively more helpful for feeling better (i.e., hedonic goal), whereas interpersonal suppression and ignoring were considered as relatively more helpful for getting work done (i.e., instrumental goal). In contrast, interpersonal reappraisal and problem-solving were considered equally effective for both goal types. In sum, although overall differences in perceived effectiveness between strategies were more pronounced than the goal-related differences, the findings nevertheless suggest that regulatory goals can shape which IER strategies are perceived as relatively more helpful.

## 3. Study 2

Study 1 examined perceived helpfulness of IER strategies for achieving hedonic vs. instrumental goals by relying on participants’ goal-specific evaluations of potential partner responses. Study 2 extended these findings by examining typical partner responses in the context of ongoing romantic relationships. Participants rated their partners’ typical IER use and how effective their partners are in making them feel better and facilitating goal achievement. As such, by correlating two independently answered questionnaires, this study allowed for a somewhat more conservative test compared to Study 1. This design allowed us to test which specific IER strategies are helpful for hedonic and instrumental goals. No specific hypotheses were preregistered for this study.

### 3.1. Materials and Methods

#### 3.1.1. Participants

Following practical considerations and guidelines in the field (Finkel et al., 2015), 190 couples (380 individuals) were recruited in the Netherlands via websites, social media, and personal networks to participate in the study. To be eligible, participants were required to be in a romantic relationship for at least four months. At intake, we excluded data from participants who failed two out of three attention checks, withdrew from the study, or had technical issues with the survey. This resulted in a total of 361 participants, of which 52% identified as women and 48% as men, with ages ranging from 18 to 75 years old (*M* = 38.7; *SD* = 14.5). Most of the participants identified as Dutch (90.0%), with the remainder of the sample identifying as EU resident (2.4%), Indonesian-Dutch (1.3%), African/Maghreb (0.3%), Surinamese (1.3%), Caribbean (1.1%), Chinese (1.1%), Spanish (non-EU, 0.3%) and other (2.4%). On average, partners had been in a relationship with each other for 12.4 years (*SD* = 12.3 years), and 82.8% of the couples were living together.

#### 3.1.2. Procedure

The study was approved by the Ethics Review Board of the Faculty of Social and Behavior and Movement Sciences of VU University, Amsterdam, the Netherlands. The data for the present study are part of a larger research project that included an intake session with questionnaires, followed by a daily diary phase and subsequent follow-up surveys. The measures relevant for our current research question were only assessed at the intake survey. Participants signed informed consent and then filled out various surveys about their relationship, emotion regulation tendencies, well-being, work life and personality. Included in this survey was a questionnaire regarding their partner’s typical IER use, and (after several other questionnaires), two items tapping into perceived partner efficacy in relation to hedonic and instrumental goal achievement. At the end of the study, participants were debriefed and financially compensated. Participants were paid up to 90 euros in total for the entire project.

#### 3.1.3. Measures

**IER Strategy Use:** Participants rated their partner’s typical use of seven different IER strategies in response to participants ‘being bothered by negative feelings’. As in Study 1, these strategies included co-rumination (“My partner tries to get me to talk over and over about what is bothering me”), interpersonal suppression (“My partner tells me not to feel bad (e.g., “don’t cry, don’t be sad, don’t worry”)”), interpersonal distraction (“My partner distracts me”), interpersonal reappraisal (“My partner tries to make me look at things from a different perspective”), interpersonal acceptance (“My partner says it’s OK to feel the way I’m feeling”), interpersonal problem-solving (“My partner gives me practical advice on how to solve the issue”), and interpersonal ignoring (“My partner ignores my feelings”). Each strategy was measured by one item and rated on a scale ranging from 1 (“not at all”) to 7 (“extremely”). Items were taken and adapted from prior studies (Heiy & Cheavens, 2014; Pauw et al., 2019; Swerdlow & Johnson, 2022), and previously used by Pauw et al. (2024).

**Perceived Partner Efficacy:** Participants first reported to which extent their partner is efficacious in making them feel better (“My partner is able to help me feel better”) and then to which extent their partner is efficacious in helping them achieve their goals (“My partner is able to help me achieve my goals”) on a 7-point Likert scale ranging from 1 (“not at all”) to 7 (“extremely”).

### 3.2. Results

We examined the effectiveness of the seven of IER strategies for each goal type using a two-level multilevel modeling approach to account for the non-independence in the data, given that participants were nested within couples. All seven IER strategies were entered simultaneously as fixed effects predictors of perceived partner efficacy, and non-independence of observations within couples was modeled using a compound symmetry covariance structure (Kenny et al., 2006). Separate models were estimated for each regulatory goal. Multicollinearity diagnostics based on the fixed-effects structure of the multilevel model indicated low levels of overlap among predictors (VIFs = 1.20–1.55). Descriptive statistics are reported in Table 2; test statistics are reported in Table 3; correlations among all variables are reported in Table S3 in the Supplementary Materials.

Regarding the hedonic goal, partners’ use of interpersonal reappraisal and acceptance positively predicted perceived partner efficacy in making participants feel better, whereas partners’ use of ignoring was a negative predictor. Partners’ use of interpersonal suppression, distraction, problem-solving and co-rumination were not significantly related to perceived efficacy of making participants feel better. Regarding the instrumental goal, interpersonal reappraisal, co-rumination, and problem-solving were significant positive predictors of perceived partner efficacy in helping participants achieve their goals. Partners’ use of interpersonal acceptance, distraction, suppression and ignoring were unrelated to perceptions of partner efficacy in fostering goal achievement.

### 3.3. Discussion

Study 2 aimed to examine the absolute effectiveness of seven IER strategies for hedonic and instrumental goals. To this end, participants rated their partner’s typical IER use and, in a separate set of questions, the extent to which they perceived their partner as efficacious in making them feel better and achieve their goals. Results showed that partners’ use of interpersonal reappraisal and acceptance were positively associated with perceiving the partner as helpful in making one feel better, while partners’ ignoring was negatively associated with perceived efficacy in improving emotional well-being. Partners’ use of interpersonal reappraisal, problem-solving and co-rumination were positively associated with perceiving the partner as generally helpful in fostering goal achievement. Taken together, these results indicate that partners’ IER behaviors are differentially linked to perceived partner efficacy for emotional versus goal-related outcomes. At the same time, some strategies (e.g., reappraisal) may be effective for both hedonic and instrumental regulatory goals.

## 4. Study 3

Study 1 assessed the perceived effectiveness of IER strategies in the context of hedonic vs. instrumental goals, and Study 2 tested associations between typical IER strategy use and general perceived partner efficacy for these goals. As such, both studies relied on cross-sectional reports of perceived helpfulness rather than on observed changes in emotional experience or goal achievement. To address this limitation, Study 3 moved beyond perceived effectiveness and examined whether partner IER predicted actual change over time in hedonic and instrumental goal outcomes in a naturalistic context. More specifically, participants reported on a personally relevant goal, allowing us to assess whether partners’ use of different IER strategies predicted changes in goal-related negative affect and progress toward that goal over time. These self-generated goals reflected personally meaningful, real-life pursuits across domains such as health, work and finances, which typically require sustained goal engagement and adaptation to setbacks, and are therefore relevant to processes of personal growth (Lord et al., 2010; Mann et al., 2013). By focusing on a self-relevant goal and tracking outcomes across a one-month period, this design provides a more ecologically valid test of whether IER strategies are associated with actual changes in both emotional experience and goal pursuit. No specific hypotheses were preregistered for this study.

### 4.1. Materials and Methods

#### 4.1.1. Participants

Participants were recruited using Prolific. Initially, 1207 participants completed both surveys. Eligibility criteria included: (1) being in a committed romantic relationship, (2) completing both phases of the study, (3) choosing a goal unrelated to one’s romantic partner, (4) remembering one’s own goal for the second phase, (5) reporting the same goal in both phases, and (6) stating that they provided honest responses in both phases. After data cleaning, 494 participants were eligible for further analyses (181 participants did not pass both honesty checks; 248 participants did not remember their goal at Time 2; 15 participants chose a goal related to the romantic partner at Time 1; 28 participants chose a goal related to the romantic partner at Time 2; for 151 participants, the goal category of Time 2 did not match the goal category of Time 1; for 90 participants, the goal description between Time 1 and Time 2 did not match up).^1^ A sensitivity power analysis indicated that this sample size provided 80% power to detect a small-to-medium effect (Cohen’s *f* = 0.17) at α = .05 in a multiple regression model with seven predictors. Participants were aged between 20 and 79 years old (*M* = 43.2, *SD* = 12.5), of which 35.6% identified as male and 63.2% as female, while 1.2% identified as “other” or preferred not to reveal. Most participants resided in the United Kingdom (98.6%; remaining 1.4% in the United States). 

#### 4.1.2. Procedure

The study was approved by the Ethics Review Board of the Faculty of Social and Behavior and Movement Sciences of VU University, Amsterdam, the Netherlands. The study was conducted in two phases, with a one-month interval between. Participants provided informed consent for each study phase individually. In the first phase, participants were asked to identify a personal goal and respond to a series of questions (including goal-related negative emotions and progress). They were reminded to make note of the goal for the second phase. In the second phase, the same participants were asked to recall the same goal and answer the same questions as at Time 1. Additionally, participants rated their partner’s use of IER strategies in response to their goal-related negative affect over the past month. Next, they answered several demographic questions, as well as an honesty check. Participants who indicated that they could not remember their original goal answered some filler items and were directed to the end of the survey. Finally, participants were thanked, debriefed and financially compensated. They were paid £0.25 for participation in the first phase, and £1 for participation in the second phase.

#### 4.1.3. Measures

**Personal Goal Selection:** In the first phase, participants were asked to choose a goal which they hoped to accomplish and that had to meet three specific criteria: (1) the goal caused them to feel negative emotions; (2) they would be able to make progress on the goal within a month by taking certain actions; and (3) the goal was not related to their romantic relationship, but to a different domain in life. Participants were asked to describe this goal and select which domain it pertained to: work (21.9%), education/school (2.4%), finances (11.1%), health (49.4%), sport (4.7%), hobbies (5.7%), relationship with partner (used to filter out responses; 0%), other relationships (1.6%), or “other” (3.2%). They were instructed to make note of the chosen goal (e.g., by a screenshot) as it would be relevant for the second phase.

In the second phase, participants were asked (1) whether they remembered their goal (yes/no); (2) to describe their goal again, and (3) which domain the goal pertained to, using the same options as in the first phase. Only those who reported the same goal domain at both time points were eligible for further analyses. The open-ended goal description questions were also used during data cleaning, whereby the researcher manually evaluated whether responses across the two phases were sufficiently similar to be considered as reflecting the same goal.

**Instrumental Goal Achievement: Goal Progress:** To assess instrumental goal achievement, participants rated their goal progress at both time points (i.e., “How much progress have you already made toward your goal?”). An index for goal progress over time was created by subtracting goal progress scores at Time 1 from goal progress scores at Time 2, meaning that higher scores reflected greater subjective goal progress over time. In addition, at Time 2, participants reported their retrospectively perceived goal progress since Time 1 (i.e., “How much progress have you made toward your goal since the last study, one month ago”?). All questions were measured using a 10-point scale ranging from 0 (“no progress at all”) to 10 (“reached my goal”).

**Hedonic Goal Achievement: Affective Improvement:** To assess hedonic goal achievement, participants reported their goal-related negative affect at both time points, allowing us to model affective improvement over time. We assessed several negative emotions commonly elicited during goal pursuit (anger, anxiety, frustration, guilt, worry, sadness, and overall negative affect; Carver, 2004). Each emotion was measured with a single item on a 7-point Likert scale (“How much does this goal trigger [emotion] in you?”) on a 7-point Likert scale (1 = not at all, 7 = completely). CFAs were generally consistent with modeling these seven items as indicators of a general negative affect factor (see Supplementary Section S2 for more details). Accordingly, an average score of negative affect was computed for each time point (α_time1_ = .85, α_time2_ = .93). An index for affective improvement was created by subtracting the average negative affect score at Time 2 from the average negative affect score at Time 1, meaning that higher scores reflected greater affective improvement over time.

**IER Strategy Use:** At Time 2, participants rated the extent to which their romantic partner had engaged in specific IER behaviors over the past month when the participant experienced negative feelings related to their goal. IER strategies were assessed using the same scale as in Study, with slight adaptations to ensure items referred specifically to “this goal” (i.e., the goal identified at Time 1 and monitored across both waves). One interpersonal suppression item was reworded based on its weak psychometric performance in Study 1. Items were presented in random order. The scale included three items for each of the seven IER strategies: interpersonal acceptance (α = .86), interpersonal reappraisal (α = .87), interpersonal problem-solving (α = .88), co-rumination (α = .72), interpersonal distraction (α = .77), interpersonal suppression (α = .53), and interpersonal ignoring (α = .83). CFAs were broadly consistent with the proposed seven-factor structure of the IER scale, although the reworded suppression item again showed consistently weak psychometric performance and was therefore excluded from the final model (see Supplementary Section S2 for more details). Accordingly, IER strategy scores were computed as the mean of their respective items, with interpersonal suppression averaged over the two retained items. Higher scores reflected greater perceived use of each strategy over the past month. For the full scale, see Supplementary Section S1.

### 4.2. Results

#### 4.2.1. Descriptive Statistics

Negative affect was higher at Time 1 (*M* = 4.40, *SD* = 1.23) than at Time 2 (*M* = 3.66, *SD* = 1.55), indicating that overall, participants experienced a decrease in goal-related negative affect. Participants reported moderate goal progress at Time 1 (*M* = 3.47, *SD* = 1.88), which increased at Time 2 (*M* = 5.24, *SD* = 2.54). This corresponds to an average increase in goal progress of 1.77 points on a 10-point Likert scale (*SD* = 2.66). Interestingly, however, the progress that was retrospectively reported by participants at Time 2 was higher (*M* = 5.26, *SD* = 2.64), suggesting a potential tendency to overestimate goal progress when reported retrospectively.

#### 4.2.2. IER Use and Hedonic and Instrumental Goal Achievement

To examine whether partners’ use of different IER strategies predicted affective improvement (hedonic goal) and goal progress (instrumental goal) over time, we ran two multiple regression analyses, one for each outcome. All IER strategies were entered simultaneously as predictors. Multicollinearity was within acceptable limits (VIFs = 1.61–3.82). Descriptive statistics and test statistics are reported in Table 4; correlations among all study variables are reported in Table S4 in the Supplementary Materials.

For hedonic goal achievement, problem-solving positively predicted significant affective improvement over time (i.e., a decrease in negative affect). In contrast, interpersonal reappraisal and co-rumination negatively predicted affective improvement (i.e., they were associated with increased negative affect over time). The remaining IER strategies did not significantly predict changes in negative affect. For instrumental goal achievement, only problem-solving predicted a significant increase in goal progress over time. No other IER strategies were associated with significant changes in goal progress. These findings were consistent when examining participants’ retrospectively perceived goal progress over the past month: Problem-solving positively predicted self-assessed goal progress (β = .32, *B* = 0.47 [0.11], *t*[485] = 4.20, *p* < .001), whereas none of the other IER strategies showed significant associations (all *p*’s >.05; see Table S5, Supplementary Section S3 for all test statistics).

### 4.3. Discussion

The results of Study 3 indicated that problem-solving was the only strategy that was associated with both improved hedonic and instrumental outcomes over time, predicting a reduction in negative affect as well as greater goal progress. In contrast, co-rumination and interpersonal reappraisal were associated with increased negative affect across the one-month period. None of the other strategies were linked to longitudinal changes in either outcome. Overall, these findings suggest that IER strategies differ in how they relate to longer-term affective improvement and goal progress.

## 5. General Discussion

### 5.1. Summary of Findings

Across three studies, we examined whether the effectiveness of interpersonal emotion regulation (IER) strategies depends on the regulatory goal—whether to help a partner feel better (hedonic) or to support goal progress (instrumental). To this end, we examined which strategies are most effective for each goal, as well as whether some strategies are relatively more effective for one goal than the other. Study 1 showed that, when asked to rate the effectiveness of different strategies for hedonic or instrumental goals, targets perceived interpersonal acceptance, reappraisal and problem-solving as most effective for both regulatory goals, whereas interpersonal ignoring was perceived as least effective for both goals. Importantly, the perceived helpfulness of IER strategies varied depending on whether targets aimed to feel better or to get work done: Partner use of interpersonal acceptance, co-rumination, and distraction was perceived as more effective for hedonic (vs. instrumental) goals, whereas interpersonal suppression and ignoring were perceived as relatively more effective for instrumental (vs. hedonic) goals. These findings suggest that people may intuitively distinguish between strategies that provide emotional relief and those that may facilitate task engagement.

Study 2 examined naturally occurring IER in close relationships and examined associations between partners’ typical IER behaviors and perceived partner efficacy in relation to hedonic and instrumental goals. Here, partners’ greater typical use of interpersonal acceptance and reappraisal and lower use of ignoring was associated with perceiving the partner as generally efficacious in making them feel better, whereas partners’ greater use of interpersonal reappraisal, problem-solving and co-rumination was associated with perceiving the partner as generally efficacious in fostering goal accomplishment. These findings partly converge with people’s goal-dependent perceptions of strategy helpfulness observed in Study 1 (e.g., the perceived effectiveness of interpersonal acceptance for hedonic goals), but also suggest that some strategies (e.g., interpersonal reappraisal) may serve both emotional and instrumental functions.

Finally, Study 3 employed a longitudinal design to assess whether the partner’s use of the seven IER strategies predicted changes in negative affect and goal progress over one month. Results revealed that only problem-solving was associated with improvements over time: People who had perceived their partner to engage in more problem-solving over the past month, reported a greater reduction in goal-related negative affect and reported greater goal progress. Notably, co-rumination and interpersonal reappraisal were associated with increases in negative affect over time. The other strategies did not predict longitudinal change.

Taken together, our findings indicate that people perceive IER strategies as differentially helpful depending on whether they are pursuing hedonic versus instrumental goals. However, these perceptions of helpfulness did not always correspond to measurable improvements in affect or goal progress over time. Across studies, a consistent pattern emerged: strategies that are perceived or experienced as helpful in the moment are not necessarily associated with longer-term improvements in emotional well-being or goal achievement.

### 5.2. Theoretical Implications

Personal growth is considered an ongoing process in which people pursue meaningful goals while adapting to setbacks and continuously changing circumstances (Bobinet et al., 2025). Close others can play a central role by supporting (or undermining) how their partner regulates their emotions in response to such challenges, which can help them persist through difficulties and remain engaged with their goals (Feeney & Collins, 2015; Fitzsimons & Finkel, 2010). Importantly, the specific interpersonal emotion regulation (IER) strategies that are used by close others appear to be differentially associated with how effectively individuals pursue goals and maintain emotional well-being.

Overall, our findings suggest that IER strategies that engage with the emotional experience or underlying situation (e.g., interpersonal acceptance, reappraisal and problem-solving) might be more effective for both instrumental and hedonic goals than strategies that promote disengagement (e.g., interpersonal suppression and ignoring). Across studies, problem-solving emerged as the strategy most consistently associated with beneficial outcomes: it was perceived as helpful for both goal types and was associated with reduced negative affect and greater goal progress over time. Because problem-solving is aimed at directly resolving obstacles that give rise to negative emotions, it may reduce distress while also facilitating goal progress (Carver & Connor-Smith, 2010; Lazarus & Folkman, 1984). Goal progress, in turn, is associated with greater well-being and enhanced goal progress, creating a positive feedback loop (Brunstein, 1993; Szumowska et al., 2025). This dual functionality of problem-solving may be particularly relevant in the context of personal growth, as pursuing meaningful goals often involves setbacks and challenges that elicit negative emotions (Benita et al., 2024; Bobinet et al., 2025). In such situations, close others may thus support both emotional well-being and goal pursuit by helping address obstacles that impede progress (cf. Feeney & Collins, 2015; Fitzsimons et al., 2015). These findings also speak to the broader literature on close relationships: although the beneficial effects of social support for individuals and relationships are well established (Feeney & Collins, 2015; Rusbult et al., 2009), less is known about which forms of support are particularly effective for different outcomes. The present work highlights the value of practical support, suggesting that problem-solving may be an especially impactful way of helping others cope with emotional challenges while making progress toward meaningful goals.

However, when stressors are less controllable, interpersonal reappraisal and acceptance may be more beneficial (cf. Troy et al., 2013). Both strategies were perceived as highly effective for hedonic goals, and partners who used them more were generally viewed as better able to help targets feel better. Nevertheless, neither strategy was associated with affective improvement over time in Study 3. This pattern aligns with prior work showing mixed effects: although interpersonal reappraisal and acceptance have been shown to downregulate negative affect (Jurkiewicz et al., 2023; MacCann et al., 2025; Rimé et al., 2020; Sahi et al., 2021, 2025), other studies show null findings (Batenburg & Das, 2014; Lepore et al., 2000; Pauw et al., 2024). One possible explanation is that interpersonal reappraisal may at times be experienced as invalidating, which undermines its effectiveness (Ford & Troy, 2019; Marigold et al., 2014; Niven et al., 2015). Interpersonal acceptance, on the other hand, validates the emotional experience without resolving the underlying problem and may therefore prolong distress (Curci & Rimé, 2012; Rimé et al., 2020).

Importantly, beyond identifying strategies that are typically more adaptive than others, our findings of Study 1 suggest that the perceived helpfulness of IER strategies depends on the regulatory goal. For example, although interpersonal suppression and ignoring were not associated with affective improvement or goal progress across studies (cf. Little et al., 2013; Pauw et al., 2024), targets rated these strategies as more helpful when pursuing instrumental goals (i.e., getting work done) than when pursuing hedonic goals (i.e., seeking emotional relief). This pattern is in line with earlier work showing that people flexibly select different strategies to regulate both their own as well as others’ emotions, depending on whether their goal is hedonic or instrumental in nature (Eldesouky & English, 2019; English et al., 2017; Pauw et al., 2019). The present findings extend this work by showing that not only regulators but also targets differentiate between IER strategies in light of their immediate goal: Whether a strategy is considered helpful depends, at least partially, on what the person is trying to achieve.

At the same time, our results provide less consistent evidence that IER strategies are associated with different emotional or goal-related outcomes depending on the regulatory goal (Studies 2 and 3). Although several strategies were perceived as effective for specific goals (e.g., interpersonal acceptance, reappraisal and distraction), only problem-solving was consistently associated with improvements in both affect and goal progress over time. A somewhat mixed pattern emerged for co-rumination across studies, with co-rumination being perceived as more helpful for hedonic than instrumental goals in Study 1, linked to perceived instrumental effectiveness in Study 2, and associated with higher negative affect over time in Study 3. These findings suggest that co-rumination may be experienced as beneficial in the moment, but is associated with impaired emotional outcomes over time, particularly when accounting for overlap with more adaptive strategies (see also Bastin et al., 2014). Importantly, this pattern suggests that strategies that are perceived as helpful for a given goal do not necessarily translate into measurable improvements in affect or goal progress over time (cf. Rimé et al., 2020). IER strategies may therefore need to be evaluated not only in terms of how helpful they feel in the moment, but also with respect to their longer-term emotional and goal-related consequences.

Taken together, our findings point to a broader implication: the effectiveness of IER is inherently context-dependent (Dixon-Gordon et al., 2015). While regulatory goals seem to shape how helpful different strategies are perceived to be, our results also suggest that they are only one of several factors shaping the consequences of IER. Whether IER attempts actually improve emotional well-being or goal progress likely depends on multiple contextual features, such as the nature and intensity of the stressor, situational demands, relational dynamics and the time frame over which effectiveness is evaluated (Horowitz et al., 2001; Nozaki & Mikolajczak, 2022; Pauw et al., 2018, 2019, 2025). Individual differences, such as personality traits and attachment style, may further shape how IER strategies are perceived and the consequences they may have (e.g., McLeod et al., 2020). The somewhat mixed pattern of findings across our studies underscores the importance of specifying what works, for whom, and under which circumstances (Doré et al., 2016). To obtain such a comprehensive understanding of IER effectiveness, future studies should examine the role of these contextual features in shaping differential outcomes over time. Such work would help clarify when specific IER strategies may foster emotional relief, sustained goal engagement, relational benefits, and ultimately, personal growth.

### 5.3. Strengths, Limitations and Future Directions

The present research is characterized by several strengths. We addressed a novel and theoretically relevant question by responding to calls for a more context-sensitive approach to interpersonal emotion regulation (Aldao, 2013; Dixon-Gordon et al., 2015). Specifically, we examined whether the effectiveness of IER strategies depends on the regulatory goal, thereby extending theoretical frameworks on emotion regulation flexibility from the intrapersonal to the interpersonal domain (Aldao & Tull, 2015; Bonanno & Burton, 2013; Kashdan & Rottenberg, 2010). In doing so, we move beyond treating IER strategies as adaptive or maladaptive and instead evaluate their effectiveness in relation the goals they might serve. Moreover, we studied this research question by using three different complementary methods. Study 1 experimentally examined goal-specific evaluations of IER strategy use by the partner, tapping into people’s perceptions of helpfulness for hedonic versus instrumental goals. Study 2 provided a more conservative test within ongoing romantic relationships by assessing whether reports of partners’ typical IER use were associated with broader perceptions of their partner’s efficacy in helping targets feel better and make goal progress. Study 3 included a more ecologically valid design by longitudinally examining whether perceived partner IER use predicted actual changes in goal-related negative affect and goal progress over one month in the context of a specific, personally relevant goal. Together, these three studies provide a multi-method examination of IER across different time scales and contexts.

Nevertheless, several limitations are worth mentioning. First, our conclusions are limited to correlational data, and constrained in generalizability by sample composition and recruitment procedures. Even though Study 3 used a longitudinal design, it is not possible to draw strong causal conclusions. Future studies could use experimental designs manipulating the regulatory goal to more directly assess causal effects (cf. Netzer et al., 2015; Pauw et al., 2019). Second, our studies relied on self-report, largely limiting our studies to perceived use and perceived effectiveness of IER. While Study 3 did test long-term changes in goal-related negative affect and goal progress, these changes remain subjective in nature. Future studies could use dyadic or observational designs incorporating both partners’ perceptions or coded behaviors, to provide a more complete picture of enacted regulatory behavior and its emotional and goal-related consequences. Moreover, Study 2 used single-item measures of strategy use and perceived efficacy to reduce participant burden within a larger project. It should be noted that reliance on single items is common when assessing ER strategy use (e.g., Blanke et al., 2020; Heiy & Cheavens, 2014; Kalokerinos et al., 2019; Koval et al., 2015), though admittedly, no research has yet established their validity. In particular, single-item measures may fail to adequately capture the heterogeneity of behaviors associated with each emotion regulation strategy. Relatedly, the interpersonal suppression subscale showed limited internal coherence across studies. The items were intentionally designed to reflect distinct components of suppression (i.e., encouraging the suppression of emotional experience, expression, and thoughts; Webb et al., 2012). However, the present findings suggest that these components may function more appropriately as distinct indicators rather than a single unified scale. Supplementary analyses examining the effectiveness of these three items separately partially support this interpretation (see Supplementary Section S3, Tables S2, S6 and S7).

Finally, our studies assessed IER strategy use across relatively broad timescales, thereby collapsing across a wide variety of situations. However, as described earlier, the effects of different regulatory strategies likely depend on a multitude of situational, contextual and personal factors (Randall & Schoebi, 2018), which the methodological designs of these studies did not allow to capture. This may help explain the partially mixed findings observed across our studies. In addition, the differential effectiveness of IER strategies across studies may to some extent reflect variation in the level of abstraction and time frame over which IER was assessed, ranging from goal-contingent evaluations (Study 1), to global impressions of partners’ typical behavior (Study 2), to retrospective reports of IER use over one month (Study 3). Future studies could use video-mediated recall to examine how regulatory processes unfold within specific interactions in real time (cf. Kuhn et al., 2017; Pauw et al., 2025). Alternatively, (dyadic) experience sampling methods would enable assessing IER use and relevant situational features closer to moments in which these processes occur in daily life (cf. Kalokerinos et al., 2017), ideally also examining its effects over time and across different outcomes.

### 5.4. Concluding Remarks

Because personal growth depends on individuals’ ability to pursue meaningful goals while adapting to emotional challenges, understanding how close others can help people regulate their emotions in ways that foster goal progress and emotional well-being is critical. The present research tested the perceived effectiveness of seven different interpersonal emotion regulation strategies in relation to two goals: downregulating negative affect and facilitating goal pursuit. Moreover, we examined whether the perceived effectiveness of IER strategies is goal-dependent, thereby extending theories of regulatory flexibility to the interpersonal domain (Bonanno & Burton, 2013; Dixon-Gordon et al., 2015; Kashdan & Rottenberg, 2010). Our findings indicate that people perceived strategies as differentially helpful for achieving goals versus feeling better. However, this differentiation was much less evident when examining longitudinal associations with affective and goal-related outcomes. Rather, problem-solving emerged as the strategy most consistently associated with beneficial outcomes across both hedonic and instrumental goals, including reductions in negative affect and greater goal progress over time. Thus, offering practical support by helping others find solutions and reduce obstacles to goal pursuit may be especially conducive to personal growth, as it appears to support both emotional well-being and goal attainment. More broadly, the lack of consistent findings across studies underscores the central role of context in shaping IER outcomes. Future research is needed to better understand when, for whom, and under which conditions specific strategies foster emotional, relational and eudaimonic well-being.

## Figures and Tables

**Table 1:** Descriptive Statistics and Pairwise Comparisons of Perceived Effectiveness of Interpersonal Emotion Regulation (IER) Strategies Within and Across Regulatory Goals (Study 1).

	Total	Hedonic Goal	Instrumental Goal	Pairwise Comparisons	
IER Strategy	*M* (*SD*)	*M* (*SD*)	*M* (*SD*)	Mean Difference (*SE*)	*p*
Interpersonal Acceptance	5.29 (1.06) ^ab^	5.44 (1.01)	5.13 (1.09)	0.31 (0.07)	<.001
Interpersonal Reappraisal	5.21 (1.04) ^a^	5.24 (1.02)	5.17 (1.06)	0.07 (0.07)	.300
Interpersonal Problem-Solving	5.34 (1.10) ^b^	5.39 (1.05)	5.29 (1.14)	0.10 (0.07)	.137
Co-Rumination	3.95 (1.23) ^c^	4.14 (1.17)	3.76 (1.25)	0.38 (0.08)	<.001
Interpersonal Distraction	4.46 (1.23) ^d^	4.76 (1.14)	4.16 (1.25)	0.60 (0.08)	<.001
Interpersonal Suppression	2.97 (1.15) ^e^	2.82 (1.12)	3.11 (1.16)	0.29 (0.07)	<.001
Interpersonal Ignoring	2.36 (1.21) ^f^	2.07 (1.06)	2.64 (1.27)	0.57 (0.07)	<.001

*Note.* Means (M) and standard deviations (SD) are based on observed data. Total means sharing a common superscript do not significantly differ at *p* < .05 (reflecting adjusted pairwise comparisons between the IER strategies averaged across both goals). Mean differences reflect within-strategy comparisons between hedonic and instrumental goals. Reported *p*-values are based on Bonferroni-corrected pairwise comparisons. Different superscripts in the Total column indicate significant differences at *p* < .05 (adjusted pairwise comparisons, averaged across goals).

**Table 2:** Descriptive Statistics: Means (*M*) and Standard Deviations (*SD*) of All Variables (Study 2).

	*M* (*SD*)
Interpersonal Acceptance	5.54 (1.52)
Interpersonal Reappraisal	5.43 (1.35)
Co-Rumination	4.06 (1.90)
Interpersonal Problem-Solving	5.43 (1.36)
Interpersonal Distraction	4.46 (1.75)
Interpersonal Suppression	3.68 (1.99)
Interpersonal Ignoring	1.79 (1.30)
Perceived Hedonic Efficacy: Affect Improvement	6.38 (0.77)
Perceived Instrumental Efficacy: Goal Achievement	6.03 (1.00)

**Table 3:** Interpersonal Emotion Regulation Strategies as Predictors of Perceived Partner Efficacy in Relation to Hedonic Goals (i.e., Affective Improvement) and Instrumental Goals (i.e., Goal Achievement; Study 2).

	Perceived Hedonic Efficacy			Perceived Instrumental Efficacy		
IER Strategy	*B* (*SE*)	*t*	*p*	*B* (*SE*)	*t*	*p*
Interpersonal Acceptance	0.09 (0.03)	3.35	<.001	−0.02 (0.04)	−0.56	.575
Interpersonal Reappraisal	0.07 (0.03)	2.21	.028	0.10 (0.05)	2.22	.027
Co-Rumination	0.04 (0.02)	1.74	.082	0.08 (0.03)	2.72	.007
Interpersonal Problem-Solving	0.03 (0.03)	1.09	.276	0.12 (0.04)	2.74	.007
Interpersonal Distraction	0.04 (0.02)	1.72	.086	0.04 (0.03)	1.21	.227
Interpersonal Suppression	−0.02 (0.02)	−1.04	.301	0.01 (0.03)	0.33	.744
Interpersonal Ignoring	−0.07 (0.03)	−2.17	.031	−0.03 (0.04)	−0.59	.557

**Table 4:** Interpersonal Emotion Regulation Strategies as Predictors of Hedonic and Instrumental Goal Progress Over Time (Study 3).

	Total	Affective Improvement (Hedonic)				Goal Progress (Instrumental)			
IER Strategy	*M* (*SD*)	β	*B* (*SE*)	*t*	*p*	β	*B* (*SE*)	*t*	*p*
Interpersonal Acceptance	4.17 (1.67)	.10	0.07 (0.06)	1.28	.202	−.01	−0.02 (0.12)	−0.14	.892
Interpersonal Reappraisal	3.93 (1.63)	−.21	−0.16 (0.07)	−2.37	.018	−.09	−0.14 (0.14)	−0.98	.330
Interpersonal Problem-Solving	3.66 (1.77)	.24	0.17 (0.05)	3.11	.002	.31	0.46 (0.11)	4.02	<.001
Co-Rumination	3.02 (1.41)	−.14	−0.13 (0.06)	−2.21	.027	−.04	−0.08 (0.12)	−0.65	.513
Interpersonal Distraction	3.16 (1.43)	<.01	<0.01 (0.05)	0.06	.951	−.07	−0.13 (0.11)	−1.27	.204
Interpersonal Suppression	3.33 (1.53)	.03	0.03 (0.04)	0.60	.549	−.01	−0.02 (0.09)	−0.17	.869
Ignoring	2.60 (1.60)	.02	0.01 (0.05)	0.25	.801	.04	0.07 (0.11)	0.64	.521

## Data Availability

Data and analysis code for Study 1 and 3, as well as analysis code for Study 2, are available on the Open Science Framework (OSF) at https://osf.io/zgqb9/overview (accessed on 1 June 2026). Due to privacy concerns (couple data), data for Study 2 are available upon request.

## Note

1 Additional attrition analyses showed that completers (*M* = 4.40, *SD* = 1.23) and non-completers (*M* = 4.29, *SD* = 1.25) did not differ significantly in baseline negative affect at Time 1, *t*(1070) = 1.53, *p* = .126. Non-completers reported slightly higher baseline goal progress (*M* = 3.95, *SD* = 2.09) than completers (*M* = 3.47, *SD* = 1.88), *t*(1070) = −4.17, *p* < .001, although the absolute difference was small on a 0–10 scale (mean difference = 0.48).

## Supplementary Materials

The following supporting information can be downloaded at: https://www.mdpi.com/article/10.3390/bs16071071/s1, Supplementary Section S1 (Additional Information on Method), Supplementary Section S2 (Confirmatory Factor Analyses on Multi-Item Scales), Supplementary Section S3 (Supplemental Analyses).

## Abbreviation

The following abbreviation is used in this manuscript:

IER	Interpersonal Emotion Regulation
