# Supplementary material for: Helping to Feel Better or Do Better? Implications of Interpersonal Emotion Regulation for Emotional Well-Being and Successful Goal Pursuit
Source: Behav Sci (Basel). 2026 Jun 30;16(7):1071. doi: 10.3390/bs16071071 (PMC13406014; doi:10.3390/bs16071071)
Supplement: Supplementary file 1 [file behavsci-16-01071-s001.zip › behavsci-4265444-supplementary.pdf]

# Supplemental Materials

Helping to Feel Better or Do Better? Implications of Interpersonal Emotion Regulation for Emotional Well-Being and Successful Goal Pursuit

## Table of Content

**Supplementary Section S1: Additional Information on Methods** .......... 3

**IER Scale Used in Study 1** .......... 3

**IER Scale Used in Study 3** .......... 5

**Supplementary Section S2: Confirmatory Factor Analyses on Multi-Item Scales** .......... 6

**Study 1** .......... 6

**Study 3** .......... 6

**Supplementary Section S3: Supplemental Analyses** .......... 8

**Study 1** .......... 8

**Table S1.** Correlations Among Perceived Effectiveness of All IER Strategies Study 1 (Scale Means). .......... 8

**Analyses for Separate Interpersonal Suppression Items** .......... 9

**Table S2.** Descriptive Statistics of Perceived Effectiveness of the Three Components of Interpersonal Suppression (Separate Items) Within and Across Regulatory Goals (Study 1). .......... 9

**Study 2** .......... 10

**Table S3.** Correlations Among Variables Study 2. .......... 10

**Study 3** .......... 11

**Table S4.** Correlations Among Variables Study 3 (Scale Means). .......... 11

**Retrospectively Perceived Goal Progress** .......... 12

**Table S5.** Interpersonal Emotion Regulation Strategies as Predictors of Retrospectively Reported Goal Progress Over Time (Study 3) .......... 12

**Analyses for Separate Interpersonal Suppression Items** .......... 13

**Table S6.** Goal Progress and Affective Improvement Predicted by the Three Components (Items) of Interpersonal Suppression (Study 3). .......... 13

**Table S7.** Goal Progress and Affective Improvement Predicted by the Three Components (Items) of Interpersonal Suppression, Controlling for All Other IER Strategies (Study 3). .......... 13

## Supplementary Section S1: Additional Information on Methods

### IER Scale Used in Study 1

GENERAL INSTRUCTIONS IER SCALE

People often try to manage (or regulate) their partner's emotions, including what their partners are feeling on the inside and what they are showing on the outside. The questions below are focused on **ways** in which your partner might try to regulate your emotions.

INSTRUCTIONS INSTRUMENTAL GOAL CONDITION

We would like to ask you to think of **moments when you're upset and want to get work done.** To what extent do you find it helpful when your partner responds in the following ways:

**When I want to get work done, I find it helpful when my partner..**

INSTRUCTIONS INSTRUMENTAL GOAL CONDITION

We would like to ask you to think of **moments when you're upset and want to feel better**. To what extent do you find it helpful when your partner responds in the following ways:

**When I want to feel better, I find it helpful when my partner..**

CO-RUMINATION

1. tries to get me to talk over and over about what is bothering me.
2. repeatedly engages me in a conversation about my negative emotions.
3. and I spend a lot of time discussing the difficulties I'm experiencing.

SUPPRESSION

4. tells me not to feel bad (e.g. "don't cry, don't be sad, don't worry").
5. encourages me to keep my emotions to myself.
6. tells me not to think about what is bothering me.

DISTRACTION

7. tries to distract me.
8. tries to direct my attention to something else.
9. brings up something fun to take my attention off my negative feelings.

RE-APPRAISAL

10. tries to make me look at things from a different perspective.
11. tries to make me think differently about the situation.
12. tries to make me look at the situation in a more positive way.

ACCEPTANCE

13. expresses that it's OK to feel the way I'm feeling.
14. expresses that it is normal to feel the way I’m feeling in such circumstances.
15. expresses that I should let myself feel the emotions and accept them.

PROBLEM-SOLVING

16. gives me practical advice on how to approach the situation.
17. helps me brainstorm about solutions to the situation.
18. helps me to make a plan.

IGNORING

19. ignores my feelings.
20. does not get involved in the situation.
21. does not respond to my emotions.

All items were rated on a 7-point Likert scale ranging from 1 (“not at all”) to 7 (“very much”).

**IER Scale Used in Study 3**

INSTRUCTIONS

Please rate the extent to which you agree with following statements about your romantic partner's behavior **in the past month** when you were bothered by negative feelings about the goal that you were trying to accomplish. How did your partner respond?

CO-RUMINATION

1. My partner tried to get me to talk over and over about what was bothering me.
2. My partner repeatedly engaged me in a conversation about my negative emotions.
3. My partner and I spent a lot of time discussing the difficulties of achieving this goal.

SUPPRESSION

7. My partner told me not to feel bad (e.g. "don't cry, don't be sad, don't worry").
8. My partner discouraged me from expressing my emotions. [This item was deleted from final scale due to low psychometric properties of the subscale.]
9. My partner told me not to think about what was bothering me.

DISTRACTION

10. My partner tried to distract me.
11. My partner tried to direct my attention to something else.
12. My partner brought up something fun to take my attention off my negative feelings.

RE-APPRAISAL

13. My partner tried to make me look at things from a different perspective.
14. My partner tried to make me think differently about my goal.
15. My partner tried to make me look at the goal in a more positive way.

ACCEPTANCE

16. My partner expressed that it's OK to feel the way I'm feeling.
17. My partner expressed that it was normal to feel the way I was feeling in such circumstances.
18. My partner expressed that I should let myself feel the emotions and accept them.

PROBLEM-SOLVING

19. My partner gave me practical advice on how to achieve my goal.
20. My partner helped me brainstorm about solutions to achieve my goal.
21. My partner helped me to make a plan on how to achieve my goal.

IGNORING

22. My partner ignored my feelings.
23. My partner did not get involved in the situation.
24. My partner did not respond to my emotions.

All items were rated on a 7-point Likert scale ranging from 1 ("not at all") to 7 ("extremely").

## Supplementary Section S2: Confirmatory Factor Analyses on Multi-Item Scales

Confirmatory factor analyses (CFA) were conducted to evaluate the measurement structure of all multi-item constructs. Analyses were estimated in R package lavaan using robust maximum likelihood estimation (MLR) and full information maximum likelihood (FIML) to handle missing data. Model fit was evaluated using standard indices, including the robust comparative fit index (CFI), Tucker–Lewis index (TLI), root mean square error of approximation (RMSEA), and standardized root mean square residual (SRMR).

For interpersonal emotion regulation (IER) strategies, a seven-factor oblique measurement model was specified, with all latent factors freely correlated. Each factor represented one theoretically derived strategy dimension (interpersonal acceptance, interpersonal reappraisal, interpersonal problem-solving, co-rumination, interpersonal distraction, interpersonal suppression, and interpersonal ignoring), with no cross-loadings or correlated residuals specified. For negative affect, a single-factor model was specified, with items assessing anger, anxiety, frustration, guilt, worry, sadness, and negative affect loading on a general negative affect factor.

### Study 1

**IER Strategies.** The seven-factor CFA demonstrated adequate overall fit to the data, $\chi^2(168) = 684.56$, $p < .001$, CFI = .92, TLI = .91, RMSEA = .06, 90% CI [.06, .06], SRMR = .07, broadly supporting the distinction between the seven theorized IER strategies. All items loaded significantly on their intended factors, with standardized factor loadings ranging from .34 to .81 (all *p*'s < .001). It should be noted, however, that latent factors corresponding to several more engaging IER strategies (particularly interpersonal acceptance, reappraisal, and problem-solving) were moderately to strongly interrelated (*r*s = .56–.77), indicating substantial shared variance among these IER strategies (see also Table S1 for correlations among observed scale means).

### Study 3

**IER Strategies.** The seven-factor CFA showed marginal fit, $\chi^2(168) = 636.66$, $p < .001$, CFI = .91, TLI = .89, RMSEA = .08, 90% CI [.07, .09], SRMR = .09. Inspection of standardized loadings and residual structure indicated that one suppression item showed consistently weak factor loading and reduced shared variance with the remaining suppression indicators. Therefore, this item was removed and interpersonal suppression was modeled using the remaining two indicators in the final CFA. The revised model demonstrated acceptable fit to the data, $\chi^2(149) = 432.79$, $p < .001$, CFI = .95, TLI = .93, RMSEA = .07, 90% CI [.07, .09], SRMR = .06, consistent with the proposed seven-factor structure of IER strategies. All items loaded significantly on their intended factors, with standardized loadings ranging from .45 to .90 (all *p*'s < .001). Similar to in Study 1, latent factors corresponding to several more engaging IER strategies (particularly interpersonal acceptance, reappraisal, problem-solving, and co-rumination) were strongly correlated (*r*s = .73–.87), suggesting substantial shared variance among these forms of interpersonal engagement (see also Table S4 for correlations among observed scale means).

**Negative Affect.** A single-factor model showed inadequate fit at both time points (Time 1: $\chi^2(14) = 239.42$, $p < .001$, CFI = .79, TLI = .69, RMSEA = .22, 90% CI [.20, .24], SRMR = .08; Time 2: $\chi^2(14) = 242.16$, $p < .001$, CFI = .93, TLI = .90, RMSEA = .16, 90% CI [.14, .19], SRMR = .04). Based on theoretical and semantic overlap among specific emotion pairs, two residual covariances were added between anger and frustration, and between anxiety and worry, reflecting shared variance beyond the general affect factor. The revised models allowing these two residual covariances showed acceptable to excellent fit (Time 1: $\chi^2(12) = 61.41$, CFI = .96, TLI = .94, RMSEA = .10, 90% CI [.08, .13], SRMR = .04; Time 2: $\chi^2(12) = 23.81$, $p = .022$, CFI = .99, TLI = .99, RMSEA = .05, 90% CI [.02, .08], SRMR = .02). Standardized factor loadings were all significant ($p$’s < .001) and ranged from .55–.81 (Time 1) and .71–.91 (Time 2), supporting a general negative affect factor, while also indicating some additional overlap between closely related emotions.

## Supplementary Section S3: Supplemental Analyses

### Study 1

**Table S1.** Correlations Among Perceived Effectiveness of All IER Strategies Study 1 (Scale Means).

| | Acceptance | Reappraisal | Problem-Solving | Co-Rumination | Distraction | Ignoring |
|---|---|---|---|---|---|---|
| Interpersonal Acceptance | | .44*** | .51*** | .38*** | .26*** | -.46*** |
| Interpersonal Reappraisal | .44*** | | .63*** | .36*** | .36*** | -.36*** |
| Interpersonal Problem-Solving | .51*** | .63*** | | .40*** | .25*** | -.47*** |
| Co-rumination | .38*** | .36*** | .40*** | | .24*** | -.28*** |
| Interpersonal Distraction | .26*** | .36*** | .25*** | .24*** | | -.14*** |
| Interpersonal Ignoring | -.46*** | -.36*** | -.47*** | -.28*** | -.14*** | |
| Interpersonal Suppression | -.09** | .15*** | .01 | .13*** | .24*** | .41*** |

*Note*. * $p < .05$; ** $p < .01$; *** $p < .001$.

**Analyses for Separate Interpersonal Suppression Items**

Given the low psychometric properties of the Interpersonal Suppression Subscale, we reran our main analyses on the three different components of Interpersonal Suppression separately (see Supplement 1 for the specific items, reflecting experiential, expressive and thought suppression). Specifically, we ran a 2 (hedonic vs instrumental condition) x 3 (suppression items) ANOVA to test whether to effect of goal condition on perceived effectiveness of the strategies varied depending on the specific type of suppression. Means and pairwise comparisons are reported in Table S2. There was a main effect of suppression type ($F$ [1.83, 1815.40] = 357.22, $p < .001$, $\eta_p^2 = .27$), with all three items differing significantly from one another (all $p$'s < .001), such that encouraging others not to feel bad (experiential suppression) was perceived as most effective, followed by encouraging others not to think of what was bothering them (thought suppression), while encouraging others to keep their emotions to themselves (expressive suppression) was perceived as least effective. Importantly, results indicated that there was no significant interaction effect, indicating that the main effect of goal condition was not moderated by the type of suppression ($F$ [1.83, 1815.40] = 1.89, $p = .155$, $\eta_p^2 = .002$). That is, encouraging others not to feel, not to express or not to think of the emotional situation were all perceived as relatively more effective in the instrumental (rather than hedonic) goal condition by targets.

**Table S2.** Descriptive Statistics of Perceived Effectiveness of the Three Components of Interpersonal Suppression (Separate Items) Within and Across Regulatory Goals (Study 1).

| | Total | Hedonic Goal | Instrumental Goal |
|---|---|---|---|
| Suppression Type | *M (SD)* | *M (SD)* | *M (SD)* |
| Experiential Suppression | 3.77 (1.70) | 3.64 (1.70) | 3.91 (1.70) |
| Expressive Suppression | 2.22 (1.45) | 2.01 (1.38) | 2.43 (1.49) |
| Thought Suppression | 2.90 (1.53) | 2.80 (1.51) | 3.00 (1.55) |

## Study 2

**Table S3.** Correlations Among Variables Study 2.

| | Affective Improvement | Goal Achievement | Acceptance | Reappraisal | Problem-Solving | Co-Rumination | Distraction | Ignoring |
|---|---|---|---|---|---|---|---|---|
| Affective Improvement | | .51*** | .35*** | .31*** | .22*** | .24*** | .23*** | -.29*** |
| Goal Achievement | .51*** | | .12* | .28*** | .28*** | .24*** | .20*** | -.13* |
| Interpersonal Acceptance | .35*** | .12* | | .39*** | .23*** | .26*** | .23*** | -.39*** |
| Interpersonal Reappraisal | .31*** | .28*** | .39*** | | .46*** | .33*** | .35*** | -.22*** |
| Interpersonal Problem-Solving | .22*** | .28*** | .23*** | .46*** | | .21*** | .33*** | -.18*** |
| Co-rumination | .24*** | .24*** | .26*** | .33*** | .21*** | | .18*** | -.27*** |
| Interpersonal Distraction | .23*** | .20*** | .23*** | .35*** | .33*** | .18*** | | -.16** |
| Interpersonal Ignoring | -.29*** | -.13* | -.39*** | -.22*** | -.18*** | -.27*** | -.16** | |
| Interpersonal Suppression | -.01 | .09 | -.03 | .16** | .15** | .09 | .34*** | .19*** |

*Note.* * $p < .05$; ** $p < .01$; *** $p < .001$. Goal Achievement reflects perceived partner efficacy in facilitating goal achievement (instrumental goal); Affective Improvement reflects perceived partner efficacy in facilitating affective improvement (hedonic goal).

**Study 3**

**Table S4.** Correlations Among Variables Study 3 (Scale Means).

| | Affective Improvement | Goal Progress | Acceptance | Reappraisal | Problem-Solving | Co-Rumination | Distraction | Ignoring |
|---|---|---|---|---|---|---|---|---|
| Affective Improvement | | .25*** | .01 | -.04 | .05 | -.05 | -.01 | .00 |
| Goal Progress | .25*** | | .03 | .04 | .15*** | .05 | -.03 | -.04 |
| Interpersonal Acceptance | .01 | .03 | | .74*** | .64*** | .63*** | .45*** | -.67*** |
| Interpersonal Reappraisal | -.04 | .04 | .74*** | | .76*** | .63*** | .56*** | -.64*** |
| Interpersonal Problem-Solving | .05 | .15*** | .64*** | .76*** | | .67*** | .42*** | -.62*** |
| Co-rumination | -.05 | .05 | .63*** | .63*** | .67*** | | .39*** | -.51*** |
| Interpersonal Distraction | -.01 | -.03 | .45*** | .56*** | .42*** | .39*** | | -.30*** |
| Interpersonal Ignoring | .00 | -.04 | -.67*** | -.64*** | -.62*** | -.51*** | -.30*** | |
| Interpersonal Suppression | .00 | -.01 | .32*** | .42*** | .28*** | .25*** | .45*** | -.18*** |

*Note*. * $p < .05$; ** $p < .01$; *** $p < .001$.

## Retrospectively Perceived Goal Progress

Below in Table S5, we report the outcomes of a multiple regression model predicting retrospectively reported goal progress since the survey one month ago (i.e., since Time 1, rated at Time 2) by all seven interpersonal emotion regulation (IER) strategies (as enacted by the partner, reported by the participant). Outcomes mirror the main findings reported in the manuscript, using a difference score between goal progress reported at Time 2-Time1: Only interpersonal problem-solving was a significant positive predictor of retrospectively perceived goal progress.

**Table S5.** Interpersonal Emotion Regulation Strategies as Predictors of Retrospectively Reported Goal Progress Over Time (Study 3)

| | Retrospective Goal Progress | | | |
|---|---|---|---|---|
| IER strategy | β | *B* (*SE*) | *t* | *p* |
| Interpersonal Acceptance | -.05 | -0.08 (0.12) | -0.70 | .486 |
| Interpersonal Reappraisal | -.10 | -0.17 (0.14) | -1.21 | .228 |
| Interpersonal Problem-Solving | .32 | 0.47 (0.11) | 4.20 | **<.001** |
| Co-Rumination | .04 | 0.08 (0.12) | 0.69 | .488 |
| Interpersonal Distraction | -.01 | -0.01 (0.10) | -0.10 | .922 |
| Interpersonal Suppression | .02 | 0.03 (0.09) | 0.32 | .747 |
| Interpersonal Ignoring | <.05 | 0.01 (0.11) | 0.05 | .962 |

**Analyses for Separate Interpersonal Suppression Items**

Given the low psychometric properties of the Interpersonal Suppression Subscale, we reran our main analyses on the three different components of Interpersonal Suppression separately (i.e., experiential, expressive and thought suppression). We tested how these three components of interpersonal suppression (i.e., the three different items) predicted goal progress and affective improvement over time (see Table S6 for test statistics), as well as how these three components predicted these same outcomes when controlling for the other six IER strategies (see Table S7 for test statistics).

**Table S6.** Goal Progress and Affective Improvement Predicted by the Three Components (Items) of Interpersonal Suppression (Study 3).

| | **Goal Progress** | | | | **Affective Improvement** | | | |
|---|---|---|---|---|---|---|---|---|
| | *B (SE)* | β *(SE)* | *t* | *p* | *B (SE)* | β *(SE)* | *t* | *p* |
| Interpersonal Experiential Suppression | -0.04 (0.07) | -.03 (0.05) | -0.60 | .549 | -0.05 (0.03) | -.08 (0.05) | -1.69 | .091 |
| Interpersonal Expressive Suppression | -0.09 (0.09) | -.04 (0.05) | -0.94 | .348 | -0.10 (0.04) | -.10 (0.05) | -2.24 | **.026** |
| Interpersonal Thought Suppression | 0.03 (0.08) | .02 (0.05) | 0.40 | .689 | 0.08 (0.04) | .10 (0.05) | 2.12 | **.035** |

**Table S7.** Goal Progress and Affective Improvement Predicted by the Three Components (Items) of Interpersonal Suppression, Controlling for All Other IER Strategies (Study 3).

| | **Goal Progress** | | | | **Affective Improvement** | | | |
|---|---|---|---|---|---|---|---|---|
| | *B (SE)* | β *(SE)* | *t* | *p* | *B (SE)* | β *(SE)* | *t* | *p* |
| Interpersonal Experiential Suppression | -0.05 (0.07) | -.04 (0.05) | -0.66 | .512 | -0.03 (0.03) | -.04 (0.05) | -0.82 | .414 |
| Interpersonal Expressive Suppression | -0.06 (0.11) | -.03 (0.05) | -0.56 | .573 | -0.10 (0.05) | -.11 (0.05) | -1.96 | **.050** |
| Interpersonal Thought Suppression | 0.05 (0.08) | .03 (0.05) | 0.57 | .570 | 0.07 (0.04) | .10 (0.05) | 1.87 | .062 |
| Interpersonal Acceptance | -0.03 (0.12) | -.02 (0.08) | -0.21 | .831 | 0.06 (0.06) | .08 (0.08) | 1.00 | .316 |
| Interpersonal Reappraisal | -0.11 (0.14) | -.07 (0.09) | -0.79 | .429 | -0.13 (0.07) | -.17 (0.09) | -1.90 | .058 |
| Interpersonal Problem-Solving | 0.45 (0.11) | .30 (0.08) | 3.95 | **<.001** | 0.16 (0.05) | .23 (0.08) | 3.00 | **.003** |
| Interpersonal Co-Rumination | -0.07 (0.12) | -.04 (0.06) | -0.58 | .565 | -0.11 (0.06) | -.13 (0.06) | -1.99 | **.047** |
| Interpersonal Distraction | -0.14 (0.11) | -.08 (0.06) | -1.31 | .190 | -0.00 (0.05) | -.00 (0.06) | -0.01 | .989 |
| Interpersonal Ignoring | 0.08 (0.12) | .05 (0.07) | 0.68 | .495 | 0.04 (0.06) | .06 (0.07) | 0.76 | .446 |
